# Potential Strategies to Improve the Effectiveness of Drug Therapy by Changing Factors Related to Tumor Microenvironment

**DOI:** 10.3389/fcell.2021.705280

**Published:** 2021-08-10

**Authors:** Dehong Cao, Xiaokaiti Naiyila, Jinze Li, Yin Huang, Zeyu Chen, Bo Chen, Jin Li, Jianbing Guo, Qiang Dong, Jianzhong Ai, Lu Yang, Liangren Liu, Qiang Wei

**Affiliations:** ^1^Department of Urology/Institute of Urology, West China Hospital, Sichuan University, Chengdu, China; ^2^West China School of Medicine, Sichuan University, Chengdu, China

**Keywords:** tumor microenvironment, cancer-associated fibroblasts, tumor-associated macrophages, drug therapy, targeted therapy

## Abstract

A tumor microenvironment (TME) is composed of various cell types and extracellular components. It contains tumor cells and is nourished by a network of blood vessels. The TME not only plays a significant role in the occurrence, development, and metastasis of tumors but also has a far-reaching impact on the effect of therapeutics. Continuous interaction between tumor cells and the environment, which is mediated by their environment, may lead to drug resistance. In this review, we focus on the key cellular components of the TME and the potential strategies to improve the effectiveness of drug therapy by changing their related factors.

## Introduction

Tumor microenvironment (TME) refers to the cellular environment in which tumor cells and cancer stem cells (CSCs) exist. It can directly promote angiogenesis, invasion, metastasis, and chronic inflammation, and help maintain the stemness of the tumor (Denton et al., 2018). Different TMEs have not only adverse effects on the occurrence of tumors but also favorable consequences for patients. The composition of TME includes local stromal cells (such as resident fibroblasts and macrophages), remotely recruited cells (such as endothelial cells), immune cells (including myeloid cells and lymphoid cells), bone marrow-derived inflammatory cells, extracellular matrix (ECM), blood vessels, and signal molecules (Del Prete et al., 2017). Among them, tumor-associated myeloid cells (TAMCs) also include five different myeloid cell groups: tumor-associated macrophages (TAMs), monocytes expressing angiopoietin-2 receptor Tie2 (Tie2 expressing monocytes or TEM), myeloid suppressor cells (MDSCs), and tumor-associated dendritic cells (Kim and Bae, 2016). Together, they surround tumor cells while being nourished by a network of blood vessels. The TME plays a key role in the occurrence, development, and metastasis of tumors. It also has a far-reaching impact on the effect of therapeutics, and recent studies have shown that targeted the TME is clinically feasible (Table 1). Non-malignant cells in the TME usually stimulate uncontrolled proliferation of cells and play a tumor-promoting function in the overall processes of carcinogenesis. In contrast, malignant cells can metastasize to healthy tissues in other parts of the body through the lymph or circulatory system (Tu et al., 2014). As TME plays a decisive role in the progress of tumor treatment, it is essential to further understand the components associated with TME in order to provide more precise treatment for different types of cancer.

**TABLE 1:** Most recent clinical trials of TME targeted therapies.

Target	Inhibitors/antibodies	Clinical trial phase	Reference
**Treg cells**			
PD-1/PD-L1	Nivolumab (PD-1 inhibitor) Pembrolizumab (PD-1 inhibitor) Durvalumab (PD- L1 inhibitor) Atezolizumab (PD- L1 inhibitor) Avelumab (PD- L1 inhibitor) Cemiplimab (PD-1 inhibitor)	FDA-approved	
CTLA4	Ipilimumab (anti-CTLA4 monoclonal antibody)	FDA-approved	
LAG-3	Relatlimab (anti-LAG-3 mAb) Eftilagimod alpha (LAG-3Ig fusion protein)	Phase I/II clinical trial Phase II clinical trial	NCT01968109 NCT02614833
OX40	MEDI6383 (OX40 agonist)	Phase I clinical trial	NCT02221960
IDO	Navoximod (IDO inhibitor) Linrodostat mesylate (IDO inhibitor)	Phase I clinical trial Phase III clinical trial	NCT02048709 NCT03661320
**CAFs**			
MMPs	Rebimastat (MMP inhibitor)	Phase II clinical trial	NCT00040755
CXCR2	Reparixin (CXCR1/2 inhibitor)	Phase II clinical trial	NCT01861054
	BMS-813160 (CXCR2 antagonist)	Phase I/II clinical trial	NCT03496662
	AMD3100 (CXCR4 Inhibitor)	Phase I/II clinical trial	Lecavalier-Barsoum et al., 2018
CXCL12/CXCR4	LY2510924 (CXCR4 antagonist)	Phase II clinical trial Phase I clinical trial Phase II clinical trial	NCT01439568 NCT01837095 NCT02826486
	Balixafortide (CXCR4 antagonist) Motixafortide (CXCR4 antagonist)		
TGF-β	GC1008 (anti-TGF-β monoclonal antibody)	Phase II clinical trial	NCT01401062
**TAMs**			
CSF-1R	PLX3397 (CSF-1R inhibitor)	Phase I/II clinical trial	NCT01596751
CSF-1R	AMG820 (anti-CSF-1R monoclonal antibody)	Phase I/II clinical trial	NCT02713529
Deplete macrophages	Zoledronate, clodronate, ibandronate	Phase III clinical trial	NCT00127205 NCT00009945
TLR7	852A (TLR7 agonist) Imiquimod (TLR7 agonist)	Phase II clinical trial	NCT00319748 NCT00899574 NCT00821964
CCR2	PF-4136309 (CCR2 inhibitor)	Phase I clinical trial	NCT01413022
**MDSCs**			
PDE-5	Tadalafil (PDE-5 inhibitors)	Phase II clinical trial	NCT00752115
iNOS and arginase	NCX4016 (Nitric oxide-releasing aspirin derivative)	Phase I clinical trial	NCT00331786
MDSC differentiation	All-trans retinoic acid Inducing	Phase II clinical trial	NCT00617409
**Hypoxia**			
Hypoxia	TH-302 (hypoxia-activated prodrug) AQ4N (hypoxia-activated prodrug)	Phase III clinical trial Phase I/II clinical trial	NCT01746979 NCT00394628
**ECM**			
Hyaluronan	PEGPH20 (recombinant hyaluronidase)	Phase II clinical trial Phase III clinical trial	NCT01839487 NCT02715804
**Tumor vasculatures**			
VEGFRs, PDGFRs, KIT	Sorafenib (tyrosine kinase inhibitor) Sunitinib (tyrosine kinase inhibitor)	FDA-approved	
DLL4	OMP21M18 (anti-DLL4 monoclonal antibody)	Phase I clinical trial	NCT01189968
Notch1	OMP52M51 (anti-Notch1 monoclonal antibody)	Phase I clinical trial	NCT01778439
γ-Secretase	MK0752 (γ-secretase inhibitor)	Phase I clinical trial	NCT00106145

*PD-1, programmed cell death-1; PD-L1, programmed death-ligand 1; CTLA4, cytotoxic T lymphocyte-associated antigen-4; LAG-3, lymphocyte activation gene-3; IDO, indoleamine 2,3-dioxygenase; CAFs, cancer-associated fibroblasts; MMPs, matrix metalloproteinases; SDF-1, stromal-derived factor 1; CXCR, chemokine (C-X-C motif) receptor; TGF-β, transforming growth factor beta; CSF-1R, stimulating factor-1 receptor; TLR7, Toll-like receptor 7; MDSC, myeloid-derived suppressor cell; PDE-5, phosphodiesterase-5-inhibitor; ECM, extracellular matrix; VEGFR, vascular endothelial growth factor receptor; PDGFR, platelet-derived growth factor receptor; DLL4, Delta-like 4.*

## Cancer Stem Cells and Tumor Microenvironment

Bonnet and Dick (1997) first confirmed the existence of CSCs in patients with acute myeloid leukemia and subsequently detected CSCs in other primary tumor tissues and cell lines (Kinugasa et al., 2014; Lau et al., 2017). CSCs refer to the subpopulations of tumor cells present in tumor masses, which are characterized by tumorigenicity and self-renewal properties (Magee et al., 2012). There is increasing evidence that CSCs play a key role in tumor recurrence, metastasis, and therapeutic resistance (Najafi et al., 2019a). TME induces the interaction between cancer cells and a variety of tissue cells. The functional characteristics of CSCs are affected by differentiated cancer cells and activated extracellular signals mediated by fibroblasts, macrophages, epithelial cells, endothelial cells, and blood cells, which provide the necessary growth elements for tumor cells and play an important role in promoting and maintaining the stemness of CSCs (Rafii et al., 2002; Byrne et al., 2005; Kopp et al., 2006; Huang et al., 2010). Recent studies have shown that in addition to changes in proto-oncogenes, the occurrence and metastasis of tumors are closely related to their microenvironment.

In the TME, cancer-associated fibroblasts (CAFs) can promote and maintain the stem cell-like properties of liver cancer cells through the IL-6/STAT3/Notch signaling pathway (Xiong et al., 2018). In contrast, TAMs activate STAT3 and the hedgehog signaling pathway by secreting milk fat globule surface growth factor 8 and IL-6, thereby affecting the self-renewal and chemotherapy resistance of CSCs (Jinushi et al., 2011). Fan et al. (2014) also found that TAMs in liver cancer promote CSC phenotypes through the induction of epithelial–mesenchymal transition (EMT) by transforming growth factor β1 (TGF-β1). Moreover, IL-6 and NO secreted by MDSCs can activate STAT3 and NOTCH signaling pathways, stimulate the expression of microRNA101 in CSCs, and promote the expression of C-terminal binding protein-2 (CtBP2). The CtBP2 protein acts as a transcriptional auxiliary inhibitor factor that can directly target the core genes of stem cells Nanog and Sox2, and ultimately lead to the enhancement of the stemness of CSCs (Cui et al., 2013; Peng et al., 2016). Remarkably, these microenvironmental factors can also maintain the dryness of CSCs through Wntβ-catenin, FGFR, and MEK signaling pathways (Borah et al., 2015; Krishnamurthy and Kurzrock, 2018; Jin, 2020). CSCs can also regulate the expression and/or secretion of cytokines such as NFAT, NF-κB, and STAT signaling pathways through SOX2 and other genes, thereby regulating TME and recruiting TAMs to create an environment for the further development of tumors (Mou et al., 2015; Zeng et al., 2018). This undoubtedly supports the close connection between CSCs and TME. Considering that CSCs play a key role in the process of tumor occurrence, development, and recurrence, the microenvironment regulation strategy for the growth of CSCs is expected to become an effective means of tumor-targeted therapy.

## Cancer-Related Fibroblasts

Cancer-associated fibroblasts are the most common type of host cells in the TME. It is now generally accepted that CAFs are a heterogeneous population with distinct functions which can serve as positive and negative regulators of tumor progression (Kalluri, 2016). Under the influence of the microenvironment, CAFs obtain an activated phenotype that is different from that of normal fibroblasts. It can promote tumor progression and regulate the composition of ECM by secreting soluble factors and interacting with other types of cells (Piccard et al., 2012). In patients with prostate cancer, CAF in the TME can promote cell proliferation and sphere formation through paracrine signals, thus promoting the growth of tumor stem cells. Studies have confirmed that the presence of a large amount of CAF in the tumor stroma is associated with poor prognosis in lung, breast, and pancreatic cancer (Räsänen and Vaheri, 2010). CAF can promote tumor progression by maintaining the continuous proliferation and growth of tumor cells at the metastatic site (Li and Wang, 2011).

### Source and Function of CAF

**FIGURE 1:**
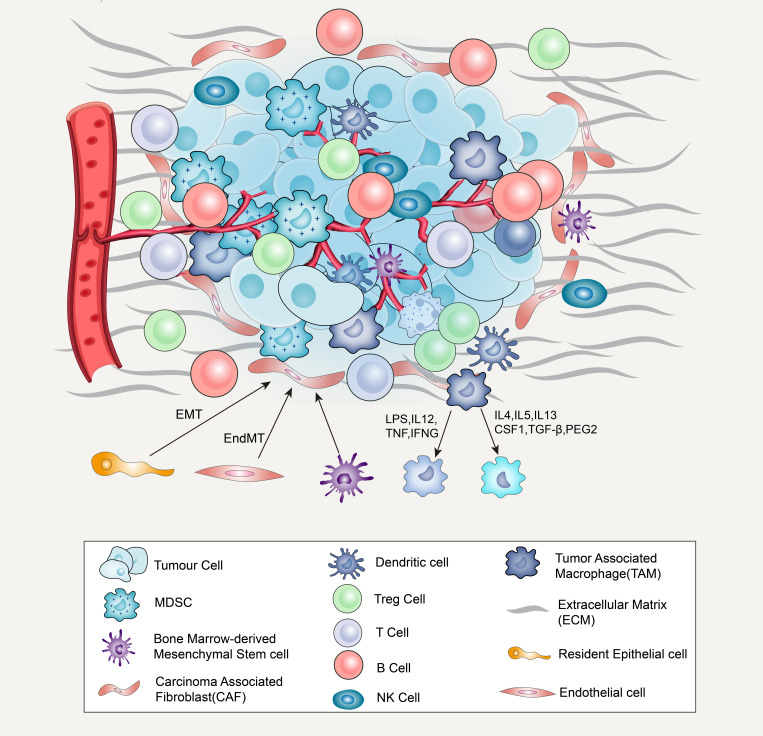
Major cellular constituents and matrix component of the TME, including cancer cells, immune cells (T-cells, B-cells, NK cells, dendritic cells, MDSCs, TAMs), CAFs and ECM. CAF derived from bone marrow mesenchymal stem cells and transform through epithelial–mesenchymal transition (EMT) or endothelial mesenchymal transition (EndMT) from resident epithelium or endothelial cells **(A)**. When macrophages are exposed to LPS, MAMPs, IL12, TNF, IFNG, or another TLR agonists, they will transition to M1-like. When exposed to IL4, IL5, IL10, IL13, CSF1, TGFβ1, and PGE2, it will transition to M2-like state **(B)**.

Most activated CAFs originate from resident fibroblasts, which can recruit and activate many growth factors and cytokines, such as transforming growth factor β, fibroblast growth factor-2, and platelet-derived growth factor (PDGF). It has been found that these growth factors and cytokines are abundant in TME (Räsänen and Vaheri, 2010). CAFs can also be derived from bone marrow mesenchymal stem cells (Figure 1), transforming from resident epithelium or endothelial cells in the tumor stroma via EMT or endothelial–mesenchymal transition (EndMT), respectively (Kidd et al., 2012). The functions of activated CAFs include the synthesis and secretion of ECM and the release of proteolytic enzymes, such as heparanase and matrix metalloproteinases (MMPs), leading to ECM remodeling (Kessenbrock et al., 2010; Wu and Dai, 2017).

Cancer-associated fibroblasts can interact with tumor cells through direct contact and can also secrete a variety of cytokines through paracrine methods to promote the occurrence and development of cancer (Kalluri, 2016; Salimifard et al., 2020). Orimo et al. (2005) have shown that CXCL12 (stromal cell-derived factor-1, SDF-1) secreted by CAFs directly stimulates tumor growth by acting through the cognate receptor, CXCR4, which is expressed by carcinoma cells. In addition, CAF-secreted vascular cell adhesion molecule-1 (VCAM-1) also promotes the proliferation, migration, and invasion of tumor cells by activating the AKT and MAPK signals of lung cancer cells (Zhou et al., 2020). Recently, Seino et al. (2018) found that CAFs can provide a Wnt-producing niche to support the *in vivo* growth of the Wnt-deficient pancreatic ductal adenocarcinoma (PDAC) organoid mode. CAFs are also an important source of growth factors and cytokines [including hepatocyte growth factor (HGF), vascular endothelial growth factor (VEGF), PDGF, etc.], which can stimulate the growth of tumor cells *in vitro* and lead to therapeutic drug resistance (Straussman et al., 2012; Erez et al., 2013; Paraiso and Smalley, 2013).

Angiogenesis in tumor tissues can provide oxygen and nutrients for tumor cell metabolism and promote tumor growth and metastasis. Many studies have shown that CAFs can release a variety of stimulating factors that promote angiogenesis and play an important role in the recruitment and proliferation of tumor vascular endothelial cells and the formation of vascular sprouts (Benyahia et al., 2017). CAFs promote angiogenesis by recruiting endothelial progenitor cells (EPCs) into carcinomas, an effect mediated in part by CXCL12 (Orimo et al., 2005). CXCL12 can activate the PI3K/AKT signaling pathway in tumor cells, upregulate the expression of VEGF in tumor tissues, and promote angiogenesis (Wen et al., 2019). VEGF activates the main signaling pathway in tumor angiogenesis by binding to its cognate receptor, VEGFR (Claesson-Welsh and Welsh, 2013). Mirkeshavarz et al. (2017) found that CAFs can secrete interleukin-6 (IL-6) and VEGF to induce angiogenesis in oral cancer, and that IL-6 can induce the secretion of VEGF in CAF cell lines. CAF can also release active growth factors from the ECM by expressing MMPs, which indirectly promotes angiogenesis (Najafi et al., 2019b) and serves as one of the sources of MMP9 (Boire et al., 2005) and MMP13 (Vosseler et al., 2009). Both these substances have been shown to release VEGF from the ECM to increase angiogenesis in tumors (Lederle et al., 2010).

Cancer-associated fibroblasts interact with tumor cells through inflammatory signals, thereby affecting tumor cell migration and invasion. The CAF-mediated CXCL12/CXCR4 axis plays a key role in tumor cell proliferation, invasion, and migration. The CXCL12/CXCR4 axis can activate the MEK/ERK, PI3K/AKT, and Wnt/β-catenin pathways to promote EMT, thereby promoting tumor invasion and metastasis (Guo et al., 2016; Zhou et al., 2019; Mortezaee, 2020). It also activates the PI3K, MAPK, and ERK1/2 signaling pathways, promotes the secretion of MMPs, reduces the adhesion of tumor cells, and increases their invasion and metastasis ability (Wu and Dai, 2017). In addition, a recent study found that CAF-secreted CXCL-1 can stimulate the migration and invasion of oral cancer cells, that there is an interdependent relationship between CAFs and cancer cells in the oral squamous carcinoma microenvironment, and that CXCL-1 can upregulate MMP-1 in CAF expression and activity (Wei et al., 2019). In addition, CAFs can change the structure and physical properties of the ECM, thereby affecting tumor cell migration and invasion (Egeblad et al., 2010).

### Drug Resistance and Targeted Therapy of CAF

The fight against drug resistance remains a major challenge in tumor treatment. CAFs mediate a variety of tumor resistance to chemotherapeutic drugs. CAFs can act on tumor cells by secreting cytokines, activating downstream signaling pathways in tumor cells, and promoting tumor resistance (Chen and Song, 2019). Studies have shown that CAFs can enhance EMT and cisplatin resistance in non-small cell lung cancer induced by transforming growth factor β by releasing high levels of IL-6, while cisplatin, in turn, promotes cancer cells to produce transforming growth factor β, resulting in CAF activation (Figure 1). CAFs can also promote chemotherapy resistance in tumor cells by secreting exosomes. Gemcitabine (GEM) is currently a chemotherapy drug that is commonly used in the treatment of pancreatic cancer. Fang et al. (2019) found that exosomal miR-106b derived from CAFs plays an important role in GEM resistance in pancreatic cancer. Recently, Zhang et al. (2020) showed that exosomal miR-522 secreted by CAFs prevents the death of cancer cells by targeting ALOX15 and blocking the accumulation of lipid-ROS. In addition, a new mechanism for obtaining gastric cancer drug resistance through the intercellular signaling pathways of USP7, hnRNPA1, exo-miR-522, and ALOX15 has been observed.

Direct ablation of CAF can promote the regression of immunogenic tumors (Feig et al., 2013), which has been explored in several recent studies, where these cells are cleared by injection of diphtheria toxin or targeting FAP-specific chimeric antigen receptor T cells; direct ablation of CAF, however, can lead to significant side effects due to lack of specificity, such as cachexia and anemia (Roberts et al., 2013; Tran et al., 2013). Because of the lack of specific markers for CAF, this method is not feasible at present, so the need to know more about the mechanism by which CAF works remains important for the development of more targeted treatments.

In a parallel study, pharmacological stimulation of the VDR was successfully performed in activated pancreatic stellate cells (PSCs). VDR is the main genomic inhibitor that is activated by PSCs. In addition, treatment with the VDR ligand calcipotriol induced matrix remodeling, which can inhibit tumor-related inflammation and fibrosis, and also improves the transport of gemcitabine to the tumor area, thus reversing chemotherapy resistance in the pancreatic ductal adenocarcinoma model (Sherman et al., 2014). Due to the complex interaction between CAF and other cells in the tumor environment, targeting some CAF subsets may cause multiple responses in the TME, which may have multiple effects depending on the individual. To eradicate cancer, the synergistic combination of CAF-targeted therapy and other effective treatments (such as immunotherapy) should also be considered.

Furthermore, the CXCL12/CXCR4 axis activates multiple signaling pathways to promote tumor cell proliferation, invasion, distant metastasis, and inhibit apoptosis. Therefore, the screening of antagonists targeting the CXCL12/CXCR4 signaling pathway is a promising target for tumor therapy. Lecavalier-Barsoum et al. (2018) found that the CXCR4 inhibitor AMD3100 can inhibit the CXCL12/CXCR4 axis in the treatment of patients with advanced disseminated high-grade serous ovarian cancer, and the combination of AMD3100 and low-dose paclitaxel can inhibit the growth of ovarian cancer cells. In osteosarcoma, AMD3100 blocks the invasion and metastasis of osteosarcoma to the lung by inhibiting the JNK and AKT pathways (Liao et al., 2015). Another CXCR4 antagonist, AMD3465, can inhibit the proliferation, colony formation, invasion, and migration of bladder cancer cells through the CXCL12/CXCR4/β-catenin axis (Zhang et al., 2018).

Micro RNA and siRNA can silence gene expression through post-transcriptional regulatory mechanisms, which may be another viable way to inhibit CXCR4 expression. In breast cancer cells, siRNA targeting CXCR4 inhibited the migration of breast cancer cells *in vitro* (Burger et al., 2011). miR-126 can also inactivate the RhoA signaling pathway in colon cancer by reducing the expression of CXCR4 and inducing a tumor suppressor effect (Yuan et al., 2016). These studies show that miRNA or siRNA targeting CXCR4 is of great significance in tumor treatment research. CTCE-9908 is composed of dimers of CXCL12, which is a competitive inhibitor of CXCL12 targeting CXCR4 and can inhibit the secretion of CXCL12 (Guo et al., 2016). Huang et al. (2009) reported that CTCE-9908 can target the CXCL12/CXCR4 axis and inhibit primary tumor growth and metastasis of breast cancer. Hassan et al. (2011) also found that CTCE-9908 combined with the anti-angiogenic agent DC101 also reduced the volume of the primary tumor and distant metastasis compared with DC101 alone. Moreover, an *in vitro* experiment proved that CTCE-9908 can inhibit the growth, invasion, and metastasis of prostate cancer (Wong et al., 2014). This evidence supports CTCE-9908 as an efficacious novel agent to prevent and treat the spread of metastatic cancer. At present, cancer treatment methods targeting CAFs and the CXCL12/CXCR4 axis are being explored and developed rapidly.

## Tumor-Associated Macrophage

Tumor-associated macrophages account for a large proportion of most malignant tumors. They promote tumor progression at different levels by promoting genetic instability, cultivating CSCs, supporting metastasis, and taming protective adaptive immunity (Mantovani et al., 2017). TAMs can be divided into M1-like and M2-like types. When macrophages are exposed to cytokines such as bacterial lipopolysaccharide (LPS), microbe-associated molecular patterns (MAMPs), IL12, TNF, interferon-γ (IFNG), or other Toll-like receptor (TLR) agonists, they will be in a pro-inflammatory and anti-tumor state, hence M1-like. When exposed to IL4, IL5, IL10, IL13, CSF1, TFGB1, and prostaglandin E2 (PGE2), it transitions from a pro-inflammatory state to an anti-inflammatory and pro-tumor state, that is, to an M2-like state (Murray et al., 2014). TAMs have a high degree of functional plasticity and can quickly adapt to changing microenvironment (Gubin et al., 2018). The necrotic and anoxic regions of the TME contain M2-like TAMs, with low fluidity, limited antigen presentation ability, and secrete a large number of tumor support factors (Wenes et al., 2016). The metabolic spectrum of TAMs is in a dynamic model, which can change with the nutritional needs of malignant tumor cells and changes in TME. It also has a far-reaching impact on the survival of TAMs, cancer progression, and tumor-targeted immune response.

The most abundant inflammatory or immune cell type is near the CAF-populated areas in the tumor stroma, indicating a close interaction between TAMs and CAF. In prostate cancer, CAF-mediated CXCL12/CXCR4 axis induces the differentiation of monocytes and possibly M1 cells into pro-tumor M2 cells. Conversely, TAMs with the M2 phenotype activate CAFs, thereby promoting tumor malignancy (Augsten et al., 2014; Comito et al., 2014). *In vitro* co-culture experiments showed that CAF-like BM-MSCs enhanced the invasiveness of TAM-like macrophages. These macrophages strongly stimulate the proliferation and invasion of CAFs, thereby synergistically promoting the development of neuroblastoma (Hashimoto et al., 2016).

Tumor-associated macrophages release TNF-α to increase MMPs secreted by tumor cells and tumor stromal cells, destroy basement membrane tissue, and promote tumor metastasis (Shuman Moss et al., 2012). TAMs also stimulate vascular endothelium to secrete VEGF by synthesizing and secreting the Wnt7b protein to regulate angiogenesis (Yeo et al., 2014). TNF-α binds to tumor necrosis factor receptor 1 (TNFR-1), activates the VEGFC/VEGFR3 pathway, and promotes lymphangiogenesis (Ran and Montgomery, 2012). In addition, transforming (TGF-β) secreted by TAMs can induce EMT of colorectal cancer cells, thereby promoting the invasion and metastasis of colorectal cancer cells (Yang et al., 2019). Notably, exosomes are one of the components in TME, which carry a variety of active substances and are the mediator of information transmission between cells (Sun et al., 2018). The exosomes of tumor cells can stimulate TAMs to secrete cytokines and enhance tumor invasion and metastasis (Trivedi et al., 2016).

### Drug Resistance and Targeted Therapy of TAM

Tumor-associated macrophages can promote tumor repair response by coordinating tissue damage and limit the anti-tumor activity of conventional chemotherapy and radiotherapy by providing a protective niche for CSCs (Mantovani et al., 2017). There is increasing evidence that macrophages play a central role in both normal and diseased tissue remodeling, including angiogenesis, basement membrane rupture, leukocyte infiltration, and immunosuppression. Therefore, TAMs have become a promising target for the development of new anticancer treatments. These methods are mainly focused on the depletion of M2-like TAMs and/or promotion of their transformation to M1-like phenotype (Cassetta and Pollard, 2018; Pradel et al., 2018). However, the effectiveness of this method may be limited by a variety of factors, such as alternative immunosuppressive cells that can compensate for TAMs, the existence of innate and acquired drug resistance mechanisms, and the emergence of strong immunosuppression after cessation of treatment (Quail and Joyce, 2017). PLX-3397 is a small-molecule inhibitor of the CSF-1 pathway. It is not only an effective tyrosine kinase inhibitor of CSF-1R, but also targeted at cKit and FLT3. Blocking CSF-1/CSF-1R can reduce TAMS and reprogramming TAMS in the TME and enhance the activation of T cells in the TME by enhancing antigen presentation. The downstream effect blocked by CSF-1/CSF-1R hinders the growth of the tumor (Zhu et al., 2014). In a mouse model of preclinical lung adenocarcinoma, PLX-3397 has been shown to change the distribution of TAMs in the TME and reduce tumor load (Cuccarese et al., 2017). In the syngeneic mouse model of BRAFV600E mutant melanoma, PLX-3397 combined with adoptive cell metastasis immunotherapy showed a decrease in TAMs (Mok et al., 2014). In similar melanoma mouse models, PLX-3397 combined with BRAF inhibitor PLX4032 significantly reduced M2 phenotypic macrophage recruitment, resulting in significant tumor growth inhibition (Ngiow et al., 2016). In addition, recent studies have shown that M2-like TAMs, which seem to be regulators of lysosomal pH, express high levels of vacuolar ATP enzymes and are expected to become a new drug target (Kuchuk et al., 2018; Liu et al., 2019). Targeting TAMs has proven to be a promising strategy, and with the deepening of preclinical development of TAM-targeted drugs and the new progress in the study of TAM mechanism, TAM-targeted therapy will become an important supplement to anticancer drugs.

## Myelogenous Suppressor Cells

Myelogenous suppressor cells (MDSCs) are a heterogeneous population composed of bone marrow progenitor cells and immature bone marrow cells (IMCs) (Gabrilovich et al., 2012). Under normal physiological conditions, IMCs produced in the bone marrow can rapidly differentiate into mature granulocytes, macrophages, or dendritic cells. In tumors and other pathological conditions, IMCs cannot normally differentiate into mature bone marrow cells under the action of cytokines, thus forming MDSCs with immunosuppressive functions, including T cell suppression and innate immune regulation (Kumar et al., 2016). In the TME, immunosuppressive cytokines such as IL-10 and TGF-β secreted by MDSCs are important factors that inhibit the anti-tumor immune response and promote tumor progression (Yaseen et al., 2020; Salminen, 2021). Studies have shown that TGF-β can inhibit the cytotoxic activity of cytotoxic T and NK cells by reducing the production of interferon-γ (IFN-γ). On the other hand, TGF-β can also inhibit the proliferation of anti-tumor immune active cells and inhibit anti-tumor immunity from the root (Salminen et al., 2018). Bone marrow mesenchymal stem cells play a role in inducing proliferation in the TME due to the interaction between cytokines and chemokines in the tumor inflammatory environment. Conversely, MDSCs can stimulate angiogenesis by producing matrix metalloproteinase 9, pro-factor 2, and VEGF, which further induces the migration of cancer cells to endothelial cells and promotes the metastasis of cancer cells (Lee et al., 2018; Yang et al., 2020).

Myelogenous suppressor cells produce high levels of inhibitory molecules, such as Arg1, reactive oxygen species (ROS), inducible nitric oxide synthase (iNOS), and prostaglandin E2 (PGE2), to directly inhibit the anti-tumor immune response induced by effector T cells (Kusmartsev et al., 2004; Gabrilovich and Nagaraj, 2009; Condamine et al., 2015; He et al., 2018). MDSCs can also inhibit the immune response by inducing regulatory T cells (Tregs), promoting the development of macrophages into M2 phenotypes, and differentiating into TAMs (Huang et al., 2006; Weber et al., 2018). Deng et al. (2017) found that MDSC-exosomes can directly accelerate the proliferation and metastasis of tumor cells by delivering miR-126a, which indicates that MDSCs have a new regulatory mechanism on tumor cells. MDSC-induced immunosuppression promotes tumor progression by promoting EMT, accelerating immune escape, and enhancing the formation of metastatic lesions (Veglia et al., 2018). Additionally, MDSCs enhance the stemness of tumor cells, promote angiogenesis by secreting IL6 and NO, and promote tumor growth, invasion, and metastasis directly or indirectly by inhibiting T cells or natural killer cells (Condamine et al., 2015, 2016).

### Drug Resistance and Targeted Therapy of MDSC

The key roles played by MDSCs in the TME show that it is necessary to target them effectively by blocking or deleting them. Although they play a key role in tumor progression, there are no FDA-approved drugs or treatments that directly target MDSCs. At present, clinical trials are underway to target the activities of iNOS Arg1 and STAT3, metabolism through CD36, transport through CXCR2, and other mechanisms for different types of cancer (Fleming et al., 2018). The antisense oligonucleotide STAT3 inhibitor AZD9150 has been used in phase 1b clinical trials of diffuse large B-cell lymphoma in combination with immune checkpoint inhibitors. Systemic administration of AZD9150 significantly decreased granulocyte MDSCs in peripheral blood mononuclear cells (PBMCs) (Reilley et al., 2018). Current targeting strategies mainly include induction of differentiation into mature cells, inhibiting its expansion and recruitment, and blocking its immune characteristics. Studies have shown that some neutralizing antibodies or inhibitors targeting chemokine systems (CXCR4, CXCR2, and CCL2) and tumor-derived factors (CSF1, GM-CSF, and IL-6) can inhibit the expansion or recruitment of MDSC (Bayne et al., 2012; Sumida et al., 2012; Highfill et al., 2014). For example, the chemokine receptor CCR5 plays a key role in the chemotaxis of MDSCs to TME (Weber et al., 2018). However, not all MDSCs express CCR5. In melanoma mice, MDSCs expressing CCR5 have stronger immunosuppressive ability than MDSCs that do not express CCR5. Blocking CCR5 can inhibit the recruitment and immunosuppressive activity of MDSCs and improve the survival rate of melanoma patients (Blattner et al., 2018).

It has been found that some drugs, such as phosphodiesterase-5 inhibitors (sildenafil, cyclooxygenase-2 inhibitors (acetylsalicylic acid and celecoxib), vardenafil and tadalafil and bardoxolone methyl, can directly block the immunosuppressive activity of MDSCs and restore T cell response (Serafini et al., 2006; Nagaraj et al., 2010; Fujita et al., 2011; Obermajer et al., 2011). Recent studies have found that MDSC-specific peptide-Fc fusion protein therapy can completely deplete MDSCs in the blood, spleen, and tumor without affecting other immune cells, and inhibit tumor growth process (Qin et al., 2014), which provides a new idea for inhibiting tumor growth *in vivo*. In patients and animal models, the failure of anti-angiogenic therapy based on inhibition of the VEGF pathway is often concomitant with an increase in the number of MDSCs or TAMs infiltrating tumor tissues (Lu-Emerson et al., 2013; Gabrusiewicz et al., 2014). Along this line of thinking, anti-VEGF therapy is thought to upregulate alternative angiogenic factors (prokinin-1 and proagonin-2) produced by myeloid cells, which may accidentally produce anti-angiogenic effects and limit tumor recurrence.

Recent studies have shown that the accumulation of MDSCs in tumors limits the effect of anti-programmed death 1 (PD1) in the treatment of rhabdomyosarcoma checkpoint blockage. Inhibition of MDSC metastasis with an anti-CXCR2 antibody can enhance the efficacy of anti-PD1 (Highfill et al., 2014). In a tumor model of tolerant mice, the removal of MDSCs with gemcitabine combined with immunotherapy can effectively break the self-tolerance and induce strong anti-tumor immunity (Ko et al., 2007). Several chemotherapeutic drugs, such as anthracyclines, platinum derivatives, and doxorubicin, can induce immunogenic cell death, thus activating an effective anti-tumor adaptive response (Kroemer et al., 2013). The chemical process for enhancing the anticancer effect of these drugs includes increasing the antigen presentation ability of dendritic cells and the subsequent CD8+ T cell response (Bracci et al., 2014). Although the current targeted therapy targeting only MDSCs does not strengthen clinical outcomes, it may play an important role in anticancer immunotherapy in the future.

## Conclusion

Most of the treatments are focused on a certain aspect of the TME. Although some of these therapeutic responses have produced positive results, a more effective way is to promote inflammatory innate immune cells, such as CD8+ T cells, and to alter many aspects of TME through a strong inflammatory response. Breakthrough drug resistance remains a major clinical challenge. The response of tumor cells to treatment depends not only on the regulation of the TME but also on the aberration of its genome. Targeted therapy cannot focus on the complete depletion of all inherent cells in the TME, as this may cause severe complications in the patient. The solution must be a complex combination, with focus on developing multidrug management that targets both tumor cells and TME to overcome resistance and improve prognosis as much as possible.

## Author Contributions

DC, XN, JL, YH, ZC, BC, JL, and JG: wrote the review article prepared and assembled the figure and table. QD, JA, LL, and QW: critically organized and revised the manuscript by incorporating significant reports. All authors contributed to the article and approved the submitted version.

## Conflict of Interest

The authors declare that the research was conducted in the absence of any commercial or financial relationships that could be construed as a potential conflict of interest.

## Publisher’s Note

All claims expressed in this article are solely those of the authors and do not necessarily represent those of their affiliated organizations, or those of the publisher, the editors and the reviewers. Any product that may be evaluated in this article, or claim that may be made by its manufacturer, is not guaranteed or endorsed by the publisher.
